# Correction: Effect of Copper Acyclovir Complexes on Herpes Simplex Virus Type 1 and Type 2 (HSV-1, HSV-2) Infection in Cultured Cells

**DOI:** 10.1155/MBD.1998.313

**Published:** 1998

**Authors:** M. Panteva, T. Varadinova, I. Turel

**Affiliations:** 1 Laboratory of Virology, Faculty of Biology Sofia University 8 Dragan Tzankov Blvd. Sofia 1421 Bulgaria; 2 University of Ljubljana Faculty of Chemistry and Chemical Technology Askerceva 5 Ljubljana 1000 Slovenia


					CORRECTION
EFFECT OF COPPER ACYCLOVIR COMPLEXES ON HERPES
SIMPLEX VIRUS TYPE 1 AND TYPE 2 (HSV-1, HSV-2) INFECTION
IN CULTURED CELLS
M. Panteva, T. Varadinova, and I. Turel2
Laboratory of Virology, Faculty of Biology, Sofia University, 8 Dragan Tzankov Blvd.,
1421 Sofia, Bulgaria
2 University of Ljubljana, Faculty of Chemistry and Chemical Technology, Askerceva 5,
1000 Ljubljana, Slovenia
Typographical errors due to the electronic transmission of the document and/or to the PC to
Macintosh translation have appeared in this paper:
pp. 19, 20, 21, 22
(g/ml should be read as #g/ml
p. 20
37(C and 4(C should be read as 37-C and 4-C, respectively
p. 21, in table 2
the viral titres, e.g. 0.79 0.09, should be read as 0.79 + 0.09
313

				

